# Phenotypic and genetic differences among group B *Streptococcus* recovered from neonates and pregnant women in Shenzhen, China: 8-year study

**DOI:** 10.1186/s12866-019-1551-2

**Published:** 2019-08-08

**Authors:** Benqing Wu, Jinzhen Su, Li Li, Weiyuan Wu, Jingsong Wu, Yuemei Lu, Wenqing Li, Jin’e Cheng, Xunhong Liang

**Affiliations:** 1 Department of Neonatal Intensive Care Unit, University of Chinese Academy of Science-Shenzhen Hospital, Shenzhen, Guangdong China; 2 Department of Laboratory Medicine, Shenzhen People’s Hospital, Second Clinical Medical College of Jinan University, Key Laboratory of Pathogenic Microorganism and Bacterial Resistance Surveillance in Shenzhen, Shenzhen, Guangdong China

**Keywords:** Group B *Streptococcus*, GBS, Resistance gene, Serotype, Sequence type

## Abstract

**Background:**

Group B *Streptococcus* (GBS) is a leading cause of early-onset disease (EOD) and late-onset disease (LOD) in infants. We sought to investigate the antibiotic susceptibility profiles, resistance genes, virulence-related genes, serotype distribution and genotypic characteristics of GBS recovered from infected or colonized neonates and pregnant women in a tertiary teaching hospital in Shenzhen, China, from 2008 to 2015.

**Results:**

High resistance rates of erythromycin (66.7–100%) were detected among early-onset GBS (EOGBS), late-onset GBS (LOGBS), neonatal colonizing GBS (NCGBS) and maternal colonizing GBS (MCGBS). 89.5–100% of four groups of GBS isolates showed resistance to tetracycline. More than 90 % of erythromycin resistant isolates of EOGBS (8/8, 100%), LOGBS (16/17, 94.1%) and NCGBS (10/11, 90.9%) harbored *ermB*, while only 9.1–17.6% harbored *mefA/E*. By contrast, 55.8% (24/43) and 62.8% (27/43) of erythromycin resistant MCGBS isolates carried *ermB* and *mefA/E* genes, respectively. The *tetO* gene was more common in tetracycline resistant EOGBS (10/11, 90.9%), LOGBS (17/17, 100%) and NCGBS (10/11, 90.9%), compared to tetracycline resistant MCGBS (12/51, 23.5%). Additionally, the *tetM* gene accounted for 90.9% (10/11), 76.5% (13/17), 45.5% (5/11) and 80.4% (41/51) of four groups of isolates, respectively. Serotype III was the most predominant in EOGBS (8/12, 66.7%) and LOGBS (15/17, 88.2%), while serotype Ib accounted for 50.0% (6/12) of NCGBS, and serotype Ia and III accounted for 45.6% (26/57) and 33.3% (19/57) of MCGBS, respectively. Sequence type 17 (ST17) was the most common in EOGBS (6/12, 50%) and LOGBS (12/17, 70.6%), while ST12 was predominant in NCGBS (5/12, 41.7%), and five STs (ST19, ST23, ST12, ST103 and ST485) accounted for 66.7% (38/57) of the MCGBS. All serotype III-ST17 isolates recovered from neonates were associated with invasive infections.

**Conclusions:**

This study shows the meaningful differences in molecular mechanisms of resistance to erythromycin and tetracycline, and the prevalence of serotypes and STs among GBS recovered from neonates and pregnant women. ST17 is predominant in neonatal invasive GBS, but rare in NCGBS and MCGBS.

**Electronic supplementary material:**

The online version of this article (10.1186/s12866-019-1551-2) contains supplementary material, which is available to authorized users.

## Background

Group B *Streptococcus* (GBS) is the primary pathogen of neonatal infections, and the common colonizer of human genitourinary and gastrointestinal tract [1]. Recently, resistance to erythromycin among invasive GBS from neonates and adults has been increasing gradually, which is mainly mediated by various macrolide resistance genes, e.g., *ermB*, *ermA*, *ermTR* and *mefA/E* [2–4]*.* In 2010, Centers for Disease Control and Prevention (CDC) issued the updated guideline for prevention of GBS infection in perinatal period, which recommended universal GBS screening for GBS colonization at 35-37 weeks’ gestation or among women with threatened preterm delivery and unknown colonization status, as well as intrapartum antimicrobial prophylaxis (IAP) in labor among those GBS colonized. A significant decrease in the incidence of early-onset GBS (EOGBS) has been reported since the implementation of IAP policies [5].

Serotyping is a traditional phenotypic method. Currently, there are 10 serotypes (Ia, Ib and II to IX) according to capsular polysaccharide (CPS) of GBS. It is believed that 40% of early-onset disease (EOD) and 60% of late-onset diseases (LOD) in the world are related with CPS III [1]. Multilocus sequence typing (MLST) has been widely applied in epidemiologic surveillance of GBS, as a relatively reliable tool for the comparison of genetic profiles of isolates recovered from various geographic areas.

Here, we have characterized GBS isolates responsible for invasive infections or colonization in newborns and pregnant women in Shenzhen, China, from 2008 to 2015. Additionally, we also investigated the status of implementation of prenatal GBS screening and IAP policies in our hospital during 8-year period.

## Results

### Antimicrobial susceptibility testing

Forty-one GBS isolates were recovered from neonates, with twelve from EOD, seventeen from LOD and twelve from colonization. Fifty-eight GBS isolates were obtained from pregnant women, including fifty-seven from colonization and one from bacteremia. As shown in Table 1, high erythromycin resistance rates (66.7–100%) were detected among the neonatal invasive/colonizing and MCGBS. Those isolates recovered from infants showed higher levels of clindamycin resistance, compared those of mothers (66.7–100% versus 49.1%). The constitutive resistance to macrolide, lincosamide, and streptogramin B (cMLS_B_ phenotype) was more common among erythromycin resistant EOGBS (8/8, 100%), LOGBS (17/17, 100%) and NCGBS (10/11, 90.9%), compared to MCGBS (28/43, 65.1%), and the inducible clindamycin resistance (iMLS_B_ phenotype) was identified in two erythromycin resistant isolates recovered from mothers. The macrolide resistance and lincosamide susceptibility (M phenotype) was more prevalent among those erythromycin resistant isolates collected from mothers (13/43, 30.2%), compared those of infants (1/36, 2.8%). Among four groups of GBS isolates, only one recovered from mother was identified as resistant to lincosamide, but susceptible to macrolide (L phenotype). Various levels of levofloxacin resistance were founded among EOGBS (3/12, 25.0%), LOGBS (2/17, 11.8%), NCGBS (3/12, 25.0%) and MCGBS (20/57, 35.1%). 89.5–100% of four groups of GBS isolates showed resistance to tetracycline. By contrast, all isolates were susceptible to penicillin and vancomycin.

**Table 1:** Susceptibility of 6 antimicrobial agents against GBS recovered from neonates and pregnant women, respectively

Antibiotics	No. of isolates of GBS recovered from neonates						No. of isolates of GBS recovered from pregnant women			
	EOD (*n* = 12)		LOD (*n* = 17)		Colonization (*n* = 12)		Colonization (*n* = 57)		Bacteremia (*n* = 1)	
	R	I	R	I	R	I	R	I	R	I
Erythromycin	8 (66.7)	0 (0)	17 (100)	0 (0)	11 (91.7)	0 (0)	43 (75.4)	0 (0)	0 (0)	0 (0)
Clindamycin	8 (66.7)	0 (0)	17 (100)	0 (0)	9 (75.0)	1 (8.3)	28 (49.1)	3 (5.3)	0 (0)	0 (0)
Tetracycline	11 (91.7)	0 (0)	17 (100)	0 (0)	11 (91.7)	0 (0)	51 (89.5)	0 (0)	1 (100)	0 (0)
Levofloxacin	3 (25.0)	0 (0)	2 (11.8)	0 (0)	3 (25.0)	0 (0)	20 (35.1)	0 (0)	0 (0)	0 (0)
Penicillin	0 (0)	0 (0)	0 (0)	0 (0)	0 (0)	0 (0)	0 (0)	0 (0)	0 (0)	0 (0)
Vancomycin	0 (0)	0 (0)	0 (0)	0 (0)	0 (0)	0 (0)	0 (0)	0 (0)	0 (0)	0 (0)

*EOD* early-onset disease, *LOD* late-onset disease, *R* resistant, *I* intermediate Parentheses refer to the percentage of isolates

### Resistance and virulence-related genes

More than 90 % of erythromycin resistant isolates of EOGBS (8/8, 100%), LOGBS (16/17, 94.1%) and NCGBS (10/11, 90.9%) harbored *ermB*, while only 9.1–17.6% harbored *mefA/E*. By contrast, 55.8% (24/43) and 62.8% (27/43) of erythromycin resistant MCGBS isolates carried *ermB* and *mefA/E* genes, respectively, and one harbored *ermTR* gene. Among clindamycin resistant or intermediate EOGBS, LOGBS, NCGBS and MCGBS, 100% (8/8), 94.1% (16/17), 100% (10/10) and 77.4% (24/31) of isolates harbored *ermB* gene, respectively, with one maternal colonizing isolate carrying *ermTR* gene, along with 37.5% (3/8), 17.6% (7/17), 20.0% (2/10) and 32.3% (10/31) of isolates carrying *lnuB* gene, respectively. Additionally, *lnuD* was detected in two clindamycin intermediate and four clindamycin susceptible MCGBS isolates.

The *tetO* gene was more common in tetracycline resistant EOGBS (10/11, 90.9%), LOGBS (17/17, 100%) and NCGBS (10/11, 90.9%), compared to tetracycline resistant MCGBS (12/51, 23.5%). Additionally, the *tetM* gene accounted for 90.9% (10/11), 76.5% (13/17), 45.5% (5/11) and 80.4% (41/51) of four groups of isolates, respectively (Table 2). Simultaneously carrying both *tetO* and *tetM* genes was more prevalent in tetracycline resistant EOGBS (9/11, 81.8%) and LOGBS (13/17, 76.5%), compared to NCGBS (5/11, 45.5%) and MCGBS isolates (3/5, 15.9%). The tetracycline resistant maternal invasive GBS isolate harbored *tetM* gene only.

**Table 2:** Distributions of resistance and virulence-related genes in GBS recovered from neonates and pregnant women, respectively

Resistance/virulence-related genes	No. of isolates			
	EOGBS	LOGBS	NCGBS	MCGBS
ERY and CLI resistance genes^*a*^				
*ermB*	4	8	8	11
*mefA/E*			1	10
*lnuB*				1
*ermB*, *mefA/E*	1	2		6^*d*^
*ermB*, *lnuB*	3	6	2	5
*mefA/E*, *lnuB*		1		2
*mefA/E*, *lnuD*				6
*ermB*, *mefA/E*, *lnuB*				2
*ermTR*, *mefA/E*				1^*d*^
TET resistance genes^*b*^				
*TetO*	1	4	5	9
*TetM*	1			37
*TetS*			1	1
*TetO*, *TetM*	8	11	4	2
*TetM*, *TetS*				1
*TetO*, *TetM*, *TetL*	1	2		1
*TetO*, *TetM*, *TetK*			1	
Virulence-related genes^*c*^				
*hylB*				4
*hylB*, *lmb*		1	2	4
*hylB*, *scpB*			1	
*hylB*, *lmb*, *scpB*	9	15	3	44
*hylB*, *lmb*, *bca*				1
*hylB*, *lmb*, *scpB*, *bac*	1		4	4
*hylB*, *lmb*, *scpB*, *bac*, *bca*	2	1	2	

*ERY* erythromycin, *CLI* clindamycin, *TET* tetracycline *EOGBS* early-onset GBS, *LOGBS* late-onset GBS, *NCGBS* neonatal colonizing GBS, *MCGBS* maternal colonizing GBS

^*a*^ 36 and 44 isolates of erythromycin and/or clindamycin resistant/intermediate GBS were detected in neonates and pregnant women, respectively

^*b*^ 39 and 51 isolates of tetracycline resistant GBS were detected in neonates and pregnant women, respectively

^*c*^ 41 and 57 isolates of GBS were detected in neonates and pregnant women, respectively

^*d*^ Inducible clindamycin resistance was detected in GBS isolate for each one, respectively

Virulence-related *hylB* gene was detected in all GBS isolates. *Lmb* and *scpB* genes accounted for 94.9% (94/99) and 87.9% (87/99) of all isolates, respectively. By contrast, *bac* gene was found in 25.0% (3/12), 5.9% (1/17), 50% (6/12) and 7.0% (4/57) of EOGBS, LOGBS, NCGBS and MCGBS isolates, respectively, as well as *bca* gene identified in 16.7% (2/12), 5.9% (1/17), 16.7% (2/12) and 1.8% (1/57) of those isolates, respectively (Table 2). The maternal invasive isolate harbored *hylB*, *lmb* and *scpB* genes simultaneously.

### Serotypes

CPS III was most predominant in EOGBS (8/12, 66.7%) and LOGBS (15/17, 88.2%), followed by Ib (3/12, 25%) and Ia (1/12, 8.3%) in EOGBS, as well as Ib (1/17, 5.9%) and V (1/17, 5.9%) in LOGBS. For NCGBS, Ib was the most prevalent serotype, accounting for 50.0% (6/12) of isolates, followed by III (3/12, 25%) and Ia (2/12, 16.7%), accompanied with one non-typeable isolate. For MCGBS, CPS Ia and III were identified in 45.6% (26/57) and 33.3% (19/57) of isolates, respectively, followed by Ib (6/57, 10.5%), II (4/57, 7.0%) and V (2/57, 3.5%). An additional file shows this in more details (see Additional file 1). The maternal invasive GBS isolate belonged to CPS Ia. None of IV, VI, VII, VIII and IX was found in the current study.

### Sequence types (STs) and clonal complexes (CCs)

**Fig. 1:**
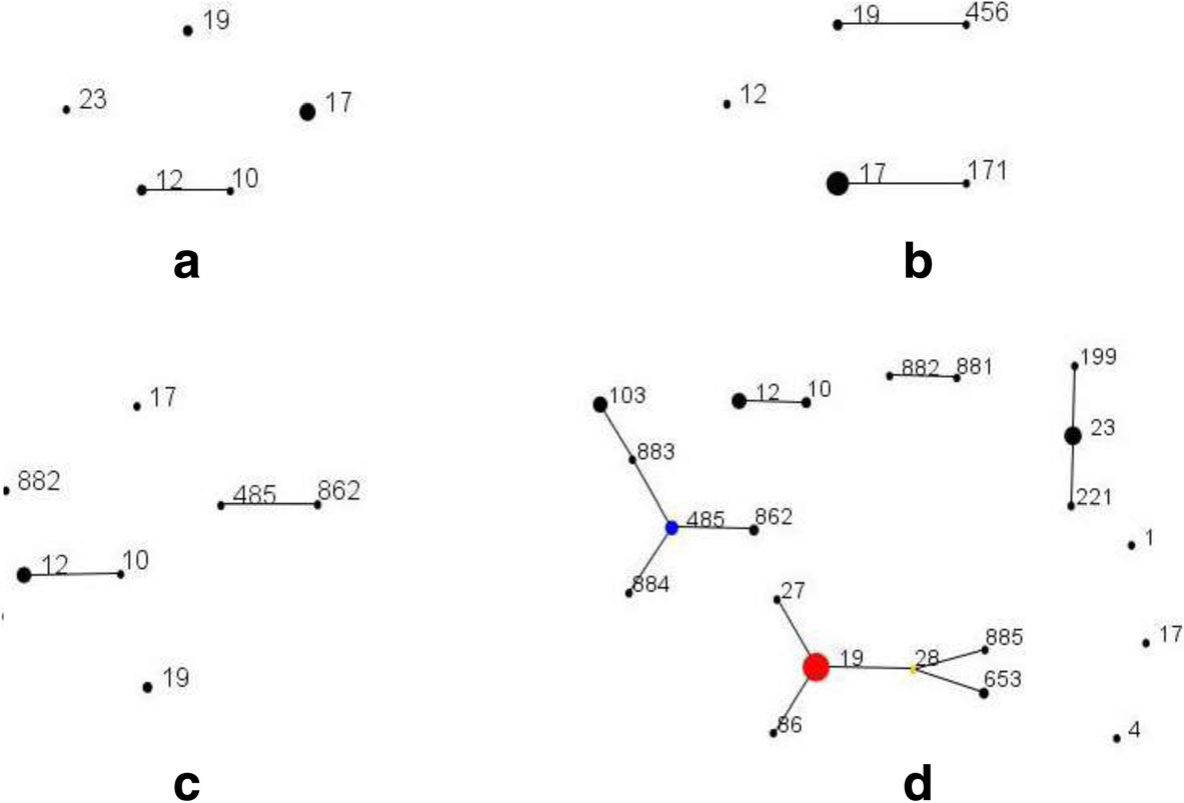
Minimum spanning tree (MST) analysis of four populations of GBS isolates according to sequence type (ST). **a** EOGBS; **b** LOGBS; **c** NCGBS; **d** MCGBS. In the MST, circles represent the STs, and the areas of each of circles indicate the prevalence of the ST in the input data. Lines are used to show the radial links from the founder to each of its single-locus variants (SLVs). The primary founder of a group is defined as the ST that differs from the largest number of other STs at only a single locus, and it is coloured blue. The user-selected primary founder is coloured red. A ST that appears to have diversified to produce multiple SLVs is called a subgroup founder, which is coloured yellow

As showed in Fig. 1, ST17 was the most common in EOGBS (6/12, 50%), followed by ST19 (2/12, 16.7%), ST12 (2/12, 16.7%), ST10 (1/12, 8.3%) and ST23 (1/12, 8.3%), and all EOGBS isolates belonged to four different CCs of CC17 (50%, 6/12), CC10 (3/12, 25%), CC19 (2/12, 16.7%) and CC23 (1/12, 8.3%). Among LOGBS, ST17 accounted for 70.6% (12/17) of isolates, followed by ST19 (2/17, 11.8%), ST12 (1/17, 5.9%), ST171 (1/17, 5.9%) and ST456 (1/17, 5.9%), and all LOGBS isolates belonged to three different CCs of CC17 (76.5%, 13/17), CC19 (3/17, 17.6%) and CC10 (1/17, 5.9%). Seven STs (ST12, ST19, ST10, ST17, ST485, ST862, and ST882) were identified in NCGBS, and ST12 was most predominant, accounting for 41.7% (5/12) of isolates. A total of twenty-one STs were identified among fifty-seven isolates of MCGBS, including ST17 in one isolate, and five STs (ST19, ST23, ST12, ST103 and ST485) accounted for 66.7% (38/57) of the isolates. An additional file shows this in more details (see Additional file 1). The maternal invasive isolate belonged to ST23.

### Associations between ST and serotype

Among neonatal invasive GBS, all ST17 isolates (*n* = 18) belonged to CPS III. By contrast, only one isolate was identified as ST17 among NCGBS, whose serotype was non-typeable. One ST17 isolate recovered from colonized pregnant woman belonged to CPS III. CPS III accounted for 91.3% (21/23) of all isolates of ST19, and CPS Ib for 92.3% (12/13) of all isolates of ST12. An additional file shows this in more details (see Additional file 1).

Eighteen CPS III-ST17 neonatal invasive isolates were recovered from septicemia (*n* = 16), bacteremia (n = 1) and omphalitis (n = 1). Sixteen out of eighteen isolates harbored *ermB* gene with resistance to erythromycin and clindamycin, and all isolates carried *tetO* gene with resistance to tetracycline (Table 3).

**Table 3:** Phenotype and molecular characterization of various serotype-ST/CC GBS recovered from infected neonates (no. of isolates)

CPS type-ST/CC	Resistance profile	ERY and CLI resistance gene	TET resistance gene	Virulence-related gene	Culture-confirmed Infection
III-ST17/CC17 (18)	ERY^R^CLI^R^ (16), TET^R^ (18)	ermB (16), mefA/E (2), lnuB (5)	tetO (18), tetM (13), tetL (1)	hylB (18), lmb (18), scpB (17)	SE (16), BA (1), OM (1)
III-ST19/CC19 (4)	ERY^R^CLI^R^ (3), TET^R^ (4)	ermB (2), mefA/E (1), lnuB (3)	tetO (3), tetM (4), tetL (1)	hylB (4), lmb (4), scpB (4)	SE (4)
III-ST171/CC17 (1)	ERY^R^CLI^R^ (1), TET^R^ (1)	ermB (1)	tetO (1), tetM (1)	hylB (1), lmb (1), scpB (1)	SE (1)
Ib-ST12/CC10 (3)	ERY^R^CLI^R^ (3), TET^R^ (3)	ermB (3), lnuB (2)	tetO (3), tetM (3)	hylB (3), lmb (3), scpB (3), bac (3), bca (2)	SE (2), OM (1)
Ib-ST10/CC10 (1)	ERY^R^CLI^R^ (1)	ermB (1)	none	hylB (1), lmb (1), scpB (1), bac (1), bca (1)	SE (1)
Ia-ST23/CC23 (1)	TET^R^ (1)	none	tetO (1), tetM (1)	hylB (1), lmb (1), scpB (1)	SE (1)
V-ST456/CC19 (1)	ERY^R^CLI^R^ (1), TET^R^ (1)	ermB (1), mefA/E (1)	tetO (1), tetM (1), tetL (1)	hylB (1), lmb (1), scpB (1)	OM (1)

*CPS* capsular polysaccharide, *ST* sequence type, *CC* clonal complex, *R* resistant, *ERY* erythromycin, *CLI* clindamycin, *TET* tetracycline, *SE* sepsis, *BA* bacteremia, *OM* omphalitis

### Clinical and biological features of GBS neonatal infections

A total of twenty-nine GBS neonatal infections (12 EOD and 17 LOD cases) were confirmed, and bloodstream infection (including sepsis and bacteremia) was the most common clinical manifestation, with 83.3% (10/12) of EOD and 94.1% (16/17) of LOD cases, respectively. Omphalitis was identified in two EOD and one LOD cases. One LOD case was simultaneously associated with sepsis and meningitis caused by GBS. The clinical signs of pneumonia and meningitis were reported in 58.3% (7/12) and 16.7% (2/12) of EOD cases, respectively, compared with 76.5% (13/17) and 52.9% (9/17) of LOD cases, respectively. Neonates with normal birth weight accounted for 100% (12/12) of EOD and 88.2% (15/17) of LOD cases, respectively. Meanwhile, full-term infants were responsible for 91.7% (11/12) of EOD and 94.1% (16/17) of LOD cases, respectively. Nine neonates with EOD and fifteen neonates with LOD received targeted treatment for GBS infections and all gained successful outcomes. Prior to obtaining the positive culture results, one neonate with EOD and one with LOD died, as well as two neonates with EOD and one with LOD were transferred to other hospitals for further therapy. An additional file shows this in more details (see Additional file 2).

### Prenatal GBS screening and IAP

The complete delivery records were obtained from mothers of early-onset cases (*n* = 9), of late-onset cases (n = 9), of colonized neonates (*n* = 11), colonized pregnant women (*n* = 47), and infected pregnant woman (n = 1), along with incomplete data for mothers of early-onset cases (*n* = 3) and late-onset cases (*n* = 8). As shown in Tables 4, 71.4% (20/28) of mothers of infected neonates and 81.8% (9/11) of mothers of colonized neonates had vaginal delivery, compared with 40.4% (19/47) of colonized pregnant women. Full term labor was more common in mothers of infected neonates (27/29, 93.1%) compared to mothers of colonized neonates (5/11, 45.5%) and colonized pregnant women (13/47, 27.7%). Premature rupture of membranes was observed in 30% (6/20) of mothers of infected neonates and 18.2% (2/11) of mothers of colonized neonates, compared with 61.7% (29/47) of colonized pregnant women. 94.4% (17/18) of mothers of infected neonates and 63.6% (7/11) of mothers of colonized neonates were not screened for GBS colonization before delivery, and 94.4% (17/18) and 72.7% (8/11) of those of them didn’t receive IAP at labor, respectively. 61.7% (29/47) of colonized pregnant women had a culture result available at labor, and 27.7% (13/47) of those of them didn’t received IAP at labor. Four mothers of infected/colonized neonates and thirty-four colonized pregnant women received incorrect IAP, and inadequate dosage and wrong timing of antibiotic administration was found within 92.1% (35/38) of cases for incorrect IAP.

**Table 4:** Prevention for perinatal GBS disease among mothers of infected/colonized neonates and colonized pregnant women

Parameter	No. of cases			
	MEOC	MLOC	MCN	CPW^*a*^
Vaginal delivery	9 (12)^*b*^	11 (16)	9 (11)	19 (47)
Cesarean delivery	3 (12)	5 (16)	2 (11)	28 (47)
Full term labor	11 (12)	16 (17)	5 (11)	13 (47)
Preterm labor	1 (12)	1 (17)	6 (11)	34 (47)
PROM ≥ 18 h	3 (11)	0 (9)	1 (11)	21 (47)
PROM < 18 h	1 (11)	2 (9)	1 (11)	8 (47)
Without PROM	7 (11)	7 (9)	9 (11)	18 (47)
Screening for GBS prior to labor	1^***c***^ (9)	0 (9)	4^*d*^ (11)	46^*e*^ (47)
Obtaining GBS culture results prior to labor	1 (9)	0 (9)	1 (11)	29 (47)
Without IAP at onset of true labor	8 (9)	9 (9)	8 (11)	13^*f*^ (47)
Correct IAP at onset of true labor^*g*^	0 (9)	0 (9)	0 (11)	0 (47)
Incorrect IAP at onset of true labor	1 (9)	0 (9)	3 (11)	34 (47)
Incorrect dosing	0 (1)	–	1 (3)	17 (34)
Incorrect dosing and timing	1 (1)	–	1 (3)	15 (34)
Non-recommended drugs^*h*^	0 (1)	–	1 (3)	2 (34)
Non-recommended drugs and incorrect timing	0 (1)	–	0 (3)	0 (34)

MEOC mother of early-onset case MLOC mother of late-onset case MCN mother of colonized neonate CPW colonized pregnant woman PROM premature rupture of membranes – none

^*a*^ Except mothers of infected/colonized neonates

^*b*^ Parentheses refer to the total of cases with available data

^*c*^ One case with threatened preterm labor

^*d*^ Four cases with preterm labor

^*e*^ Including 34 cases of preterm labor

^*f*^ Including 10 cases with unknown GBS status at the onset of labor

^*g*^ According to the 2010 guidelines recommended by CDC

^*h*^ Including erythromycin

## Discussion

Resistance to erythromycin in neonatal invasive GBS has been reported worldwide. Martins ER et al. [2] reported that erythromycin resistance in GBS recovered from neonatal invasive infections in Portugal increased from < 10% in 2005–2008 to > 30% in 2014, that was mainly associated with the presence of *ermB* (68.6%), *ermTR* (20.0%) and *mefE* (11.4%) genes. In France, the overall rate of erythromycin resistance was 16.7% in neonatal invasive GBS, increasing from 2007 to 2012, and resistance to erythromycin was mostly mediated by *ermB* (50%), *ermA* (21%) and *mef* (29%) [3]. In the current study, 66.7–100% of neonatal invasive/colonizing GBS isolates showed resistance to erythromycin and clindamycin, which was mainly mediated by *ermB* gene (> 90%), accompanied by *mefA/E* (9.1–17.6%) and *lnuB* genes (17.6–37.5%). High resistance to erythromycin in MCGBS was also observed in several studies. Lu et al. [6] reported a high resistance rate of erythromycin (66.2%) among MCGBS isolates recovered from a teaching hospital in Beijing from 2011 to 2013, which was mainly associated with the *ermB* (44.4%) genes, followed by the *ermTR* (23.3%) and *mefA/E* (22.6%) genes. The study of Teatero S et al. [7] showed that 36% of colonizing GBS isolates were resistant to erythromycin, which were recovered from healthy pregnant women in metropolitan Toronto, Canada in 2014. In the present study, MCGBS showed high resistance to erythromycin (43/57, 75.4%) and clindamycin (28/57, 49.1%). Of note, *mefA/E* and *ermB* genes were detected in 62.8% (27/43) and 55.8% (24/43) of erythromycin resistant isolates, respectively, as well as *ermB* and *lnuB* genes were identified in 77.4% (24/31) and 32.3% (10/31) of clindamycin resistant isolates, respectively. Interestingly, our study demonstrates there are meaningful differences for *ermB* and *mefA/E* for those isolates recovered for infants, compared those of mothers (> 90% versus 55.8%) and (17.6% versus 62.8%), respectively, indicating that different erythromycin resistance mechanisms being in the neonatal invasive/colonizing GBS and MCGBS in our hospital. To date, *lnuB* has been reported in GBS in a few studies [8], in contrast, other *lnu* genes have been rarely identified in GBS, although *lnuC* has been reported in this species in a study [9]. In contrast with *lnuA* in *Staphylococcus aureus* and *lnuB* in GBS, both of which confer resistance to clindamycin, *lnuD* in *Streptococcus uberis* confers resistance to lincomycin but not to clindamycin. The reason for the difference in the resistance phenotypes remains unexplained [10]. Our study showed the similar result that *lnuD* was detected only in two clindamycin intermediate and four clindamycin susceptible MCGBS isolates. To the best of our knowledge, this is the first time that the *lnuD* gene has been reported among GBS isolates recovered from pregnant women in China. In addition, neither *erm* nor *lnu* gene was detected in one clindamycin resistant isolate recovered from colonized pregnant women, though several attempts were conducted.

The present study showed that more than 90 % of tetracycline resistant isolates recovered from infants harbored *tetO*, while only 23.5% of those from mothers harbored it. Additionally, *tetM* was more prevalent in tetracycline resistant EOGBS, LOGBS and MCGBS, compared to NCGBS (76.5–90.9% versus 45.5%). It suggests that there are significant differences for the prevalence of tetO and tetM among four groups of tetracycline resistant GBS isolates.

The *bac* gene encodes the β-C protein, which is associated with epithelial cell invasion and resistance to phagocyte clearance, whereas, the *bca* gene encodes the α-C protein, which is associated with epithelial cell adherence [11]. In our study, higher occurrence of *bac* and *bca* genes were observed for those isolates recovered from infants, compared those from colonized mothers (24.4% versus 7.0%) and (12.2% versus 1.8%), respectively. Of noted, the differences for the occurrence of *bac* and *bca* genes were strongly associated with the serotypes of isolates. Our study demonstrated that the *bac* gene was only detected in the CPS Ib isolates recovered from infants (10/10, 100%) and from colonized mothers (4/6, 66.7%), as well as *bca* gene identified mainly in the CPS Ib isolates recovered from infants (5/10, 50%) and occasionally in the CPS Ia isolates from colonized mother (1/26, 3.8%). Our findings are agreement with the results of Lindahl’ study [12]. Interestingly, 50% (5/10) of CPS Ib isolates collected from infants harbored simultaneously both *bac* and *bac* genes, compared none of those from colonized mothers. The reason is not yet known.

As reported by Madrid L et al. [13], five serotypes (Ia, Ib, II, III and V) accounted for 97% of neonatal invasive GBS isolates in all regions with serotype data, according to a meta-analysis of serotype prevalence. CPS III was the most common serotype worldwide and nearly half (47%) of EOGBS cases and 73.0% LOGBS cases were caused by it. In our study, CPS III was responsible for 66.7% (8/12) of EOGBS cases and 88.2% (15/17) of LOGBS cases. Interestingly, CPS Ib accounted for 50.0% (6/12) of NCGBS isolates in our study, followed by III and Ia. Similarly, five serotypes (Ia, Ib, II, III and V) have been estimated to encompass approximately 98% of the serotypes identified during maternal colonization, according to a recent meta-analysis of maternal colonization [14]. In the research of Lu et al. [6], CPS III was identified in 41.8% of maternal colonizing isolates, followed by Ia (21.4%), V (14.9%), Ib (11.9%) and II (7.0%). In the study of Teatero S et al. [7], the most frequently identified serotypes were III (25%), Ia (23%), and V (19%) among the maternal colonizing isolates. In the present study, Ia (26/57, 45.6%) and III (19/57, 33.3%) were the most common serotypes among MCGBS, and other serotypes (Ib, II and V) accounted for 21.1% (12/57) of the remaining isolates.

Associations between ST and serotype have been reported in the literature, with some showing a strong correlation [14]. In the current study, 95% (19/20) of ST17 and 91.3% (21/23) of ST19 GBS isolates represented CPS III, and 92.3% (12/13) of ST12 isolates represented Ib. Several studies showed the correlation between CPS III-ST17 (CC17) GBS and neonatal invasive infection [2, 3, 14]. As reported by Shabayek [15], a major clone responsible for a large proportion of invasive neonatal infections are the CC17 strains mostly belonging serotype III. The CC17 strains are reported to be hypervirulent accounting for more than 80% of the GBS late-onset neonatal infections. Our study showed the similar results, CPS III-CC17 isolates accounted for 65.5% (19/29) invasive neonatal infections, however, none of twelve and one of fifty-seven (1.8%) isolates recovered from neonatal and maternal colonization, respectively, belonged to CPS III-CC17. It should be noted that the low overall incidence of invasive GBS disease in infants (data available in the subsequent discussions) is probably associated with the low colonization rates of CPS III-CC17 strains in neonates and pregnant women in our hospital.

A significant reduction in the incidence of EOGBS has been reported since the introduction of universal GBS screening and IAP policies [5, 16, 17]. In our hospital, neither national nor local IAP policy is available presently, only those pregnant women with threatened preterm delivery are required for GBS culture of vaginal or cervix swabs, and receive empirical or targeted antibiotic treatments other than IAP. In the present study, 71.4% (20/28) and 93.1% (27/29) of mothers of infected neonates had vaginal delivery and full-term labor, respectively. 94.4% (17/18) of mothers of infected neonates received neither antenatal GBS screening nor IAP at labor. These results indicate the importance of the implementation of the universal antenatal GBS screening and the IAP policies among pregnant women with full term labor and vaginal delivery. Of note, LOD accounted for 58.6% (17/29) of neonatal invasive infections in our study, which is still hard to prevent by present IAP policies. It is an urgent need to develop an efficacious maternal GBS vaccine against LOD.

In our study, the low incidence of EOGBS and LOGBS among pre-term and very pre-term/low birth weight neonates was found, and the reason remains unknown. In our hospital, more medical cares are given to the pregnant women with threatened preterm delivery, including the screening for GBS and empirical or targeted antibiotic treatments prior to labor, in spite of the absence of local IAP policy. We consider it important to reduce the incidence of EOGBS among pre-term and very pre-term/low birth weight neonates in our hospital. Also, the empirical treatments with broad-spectrum antibiotics for the complicates among pre-term and very pre-term/low birth weight neonates at the early stage of pre-term neonatal growth is common in our NICU, which could be useful to decrease the incidence of LOGBS among this population. Guan et al. investigated the epidemiology of invasive GBS collected from three large urban tertiary hospitals in South China from Jan 2011 to Dec 2014, and reported the overall and the EOGBS incidence of 0.55, 0.39 per 1000 live births, respectively [18]. In present study, with the data provided by the statistical department of our hospital, total 18 cases (9 EOGBS and 9 LOGBS) were identified from 54,192 live births born in our hospital from 2008 to 2015, giving the overall incidence of 0.33 per 1000 live births, along with the EOGBS and LOGBS incidence of 0.17 per 1000 live births, respectively. Both the overall and EOGBS incidence in our study was markedly lower than the global average estimation (0.49, 0.41 per 1000 live births, respectively) and those reported in the study from Guan et al. [13, 18]. The reason remains unclear.

The present study has a limitation of small scale of GBS isolates, with the lack of pre-term neonatal and maternal invasive GBS isolates. To the best of our knowledge, data on the epidemiology of neonatal GBS infection and bacteria resistance in China are currently very limited. Shenzhen is a rapidly growing city of immigrant from all over the country, with a large of population about thirteen million people. Shenzhen People’s Hospital is a Third-level center hospital and one of the largest urban tertiary hospitals in Shenzhen, and receives many transfers from other urban, district and rural hospitals. Our findings were representative of a large population of China. However, it should be noted that a national prospective observational study is urgently needed to investigate the incidence of invasive GBS disease in infants, antimicrobial resistance, the common resistance genes, and the distribution of serotypes and genotypes among invasive/ colonizing GBS recovered from neonates and pregnant women.

## Conclusions

This study shows the meaningful differences in molecular mechanisms of resistance to erythromycin and tetracycline, and the prevalence of serotypes and STs among GBS recovered from neonates and pregnant women. ST17 is predominant in neonatal invasive GBS, but rare in NCGBS and MCGBS.

## Methods

### Bacterial isolates

Shenzhen People’s Hospital is a 2500-bed tertiary teaching hospital in Shenzhen, China. In the current study, a total of 99 non-duplicate GBS clinical isolates were recovered from 41 neonates and 58 pregnant women (including four mother-neonate pairs) in this hospital from 2008 to 2015 (2008, *n* = 4; 2009, *n* = 3; 2010, *n* = 5; 2011, *n* = 7; 2012, *n* = 21; 2013, *n* = 20; 2014, *n* = 14; 2015, *n* = 25). In 2012, the number of beds in neonatal intensive care unit (NICU) increased from 60 to 100, indicating the significant increase of inpatients. Neonatal GBS isolated from blood (*n* = 26), umbilical secretion (n = 3), pharynx swabs (*n* = 11) and endotracheal aspirate (n = 1); Maternal GBS isolated from cervical secretion (*n* = 53), vaginal secretion (n = 2), urine (n = 2) and blood (n = 1). All isolates were identified as GBS by Vitek 2 system (bioMerieux), since 2014, by Vitek MS system (bioMerieux), along with phenotypic and manual biochemical tests.

### Serotyping and antimicrobial susceptibility testing

Capsular serotyping was carried out by a latex agglutination assay with the ImmuLex™ Strep-B kit (Statens Serum Institute, Copenhagen, Denmark), including 10 antibodies of GBS capsular polysaccharide (CPS Ia, Ib, II to IX), according to the manufacturer’s instructions.

All GBS isolates were tested for susceptibility to erythromycin, clindamycin, tetracycline, levofloxacin, penicillin, and vancomycin by using the E-test method according to the Clinical and Laboratory Standards Institute (CLSI) guidelines. Inducible clindamycin resistance (iMLS_B_) was tested by disk diffusion (D-zone test) with erythromycin and clindamycin [19].

### Resistance and virulence-related genes

DNA templates were extracted by the Lysis buffer for microorganism to direct Polymerase chain reaction (PCR) (TaKaRa, Japan). PCR amplified erythromycin and clindamycin resistance genes (*ermA*, *ermB*, *ermC*, *ermT*, *ermTR*, *mefA/E*, *lnuA*, *lnuB*, *lnuC*, *lnuD*, *lnuE* and *lnuG*), tetracycline resistance genes (*tetK*, *tetL*, *tetS*, *tetM*, *tetO* and *tetT*), and virulence-related genes (*bac*, *bca*, *lmb*, *hylb* and *scpB*) as described previously [20–27].

### MLST

MLST was performed by sequencing seven housekeeping genes (*adhP*, *pheS*, *atr*, *glnA*, *sdhA*, *glcK* and *tkt*) of GBS as described previously [28]. Alleles and sequence types (STs) were identified by using the *S. agalactiae* MLST database (http://pubmlst.org/sagalactiae/). The new alleles and STs were deposited at the *S. agalactiae* MLST database. The eBURSTv3 software (http://eburst.mlst.net) was used to display the relationships between STs, analyze clonal complexes (CCs), and create a minimum spanning tree. CCs were defined at the single locus-variant (SLV) level.

### Investigation of medical records

Medical records of GBS infected/colonized neonates and their mothers, as well as GBS infected/colonized pregnant women were investigated. The invasive infection was confirmed by the positive culture of GBS isolated from a normally sterile site (e.g. blood and cerebrospinal fluid) or an infected site (e.g. umbilicus) in our cases, along with infection-related symptoms and signs, as well as laboratory examinations. The sole positive culture of GBS isolated from a non-sterile or non-infected site (e.g. pharynx, trachea, urinary tract, cervix, and vagina), without consistent clinical findings, was defined as GBS colonization. Infection occurring in infants less than 7 days old, and that occurring in infants 7 to 89 days old, was defined as EOD and LOD [3, 5], respectively.

## Additional files

Additional file 1: **Table S1.** The details of serotypes and genotypic characteristics of GBS recovered from neonates and pregnant women, respectively. The CPS types, sequence types, clonal complexes, and predominant CPS type-STs were listed. (DOCX 15 kb)

Additional file 2: **Table S2.** The details of Clinical and biological characteristics of GBS neonatal infections. The sex, age, birth weight, gestational age, infections confirmed by culture, clinical signs of infection, targeted and empirical antibiotic treatment, and outcomes were listed. (DOCX 16 kb)

## Data Availability

The datasets used and/or analyzed during the current study are available from the corresponding author on reasonable request.

## Abbreviations

CCs: Clonal complexes
CDC: Centers for Disease Control and Prevention
CLSI: Clinical and Laboratory Standards Institute
cMLS_B_: Constitutive resistance to macrolide, lincosamide, and streptogramin B
CPS: Capsular polysaccharide
EOD: Early-onset disease
EOGBS: Early-onset GBS
GBS: Group B *Streptococcus*
IAP: Intrapartum antimicrobial prophylaxis
iMLS_B_: Inducible clindamycin resistance
LOD: Late-onset disease
LOGBS: Late-onset GBS
MCGBS: Maternal colonizing GBS
MLST: Multilocus sequence typing
NCGBS: Neonatal colonizing GBS
PCR: Polymerase chain reaction
STs: Sequence types
