# Supplementary material for: Phenotypic and genetic differences among group B Streptococcus recovered from neonates and pregnant women in Shenzhen, China: 8-year study
Source: BMC Microbiol. 2019 Aug 8;19:185. doi: 10.1186/s12866-019-1551-2 (PMC6688368; doi:10.1186/s12866-019-1551-2)
Supplement: Supplementary file 1 — Table S1. The details of serotypes and genotypic characteristics of GBS recovered from neonates and pregnant women, respectively. The CPS types, sequence types, clonal complexes, and predominant CPS type-STs were listed. (DOCX 15 kb) [file 12866_2019_1551_MOESM1_ESM.docx]

**Supplementary data**

**Table S1. Serotypes and genotypic characteristics of GBS recovered from neonates and pregnant women, respectively.**

| **GBS source** | **CPS type** | **Sequence type** | **Clonal complex** | **Predominant CPS type-ST** |
| --- | --- | --- | --- | --- |
| **Neonates** |  |  |  |  |
| **EOD (12)** | **III (8), Ib (3), Ia (1)** | **ST17 (6), ST19 (2), ST12 (2), ST10 (1), ST23 (1)** | **CC17 (6), CC10 (3), CC19 (2), CC23 (1)** | **III-ST17 (6), III-ST19 (2), Ib-ST12 (2)** |
| **LOD (17)** | **III (15), Ib (1), V (1)** | **ST17 (12), ST19 (2), ST12 (1), ST171 (1), ST456 (1)** | **CC17 (13), CC19 (3), CC10 (1)** | **III-ST17 (12), III-ST19 (2), Ib-ST12 (1)** |
| **Colonization (12)** | **Ib (6), III (3), Ia (2), NT (1)** | **ST12 (5), ST19 (2), ST10 (1), ST17 (1), ST485 (1), ST862 (1), ST882 (1)** | **CC10 (6), CC19 (2), CC485 (2), CC17 (1), CC881 (1)** | **Ib-ST12 (5), III-ST19 (2)** |
| **Pregnant women** |  |  |  |  |
| **Bacteremia (1)** | **Ia (1)** | **ST23 (1)** | **CC23 (1)** |  |
| **Colonization (57)** | **Ia (26), III (19), Ib (6), II (4), V (2)** | **ST19 (17), ST23 (7), ST12 (5), ST103 (5), ST485 (4), ST10 (2), ST653 (2), ST862 (2), others (13)^a^** | **CC19 (23), CC23 (9), CC485 (7), CC10 (7), CC103 (6), CC881 (2), others (3)*^b^*** | **III-ST19 (15), Ia-ST23 (7), Ia-ST103 (5), Ib-ST12 (4)** |

CPS capsular polysaccharide CC clonal complexes EOD early-onset disease LOD late-onset disease ST sequence type NT non-typeable. Parentheses refer to the no. of isolates.

*a* Including ST1, ST4, ST17, ST27, ST28, ST86, ST199, ST221, ST881, ST882, ST883, ST884, ST885 for each one.

*b* Including CC1(1), CC4(1), CC17(1) for each one.
