# Supplementary material for: Phenotypic and genetic differences among group B Streptococcus recovered from neonates and pregnant women in Shenzhen, China: 8-year study
Source: BMC Microbiol. 2019 Aug 8;19:185. doi: 10.1186/s12866-019-1551-2 (PMC6688368; doi:10.1186/s12866-019-1551-2)
Supplement: Supplementary file 2 — Table S2. The details of Clinical and biological characteristics of GBS neonatal infections. The sex, age, birth weight, gestational age, infections confirmed by culture, clinical signs of infection, targeted and empirical antibiotic treatment, and outcomes were listed. (DOCX 16 kb) [file 12866_2019_1551_MOESM2_ESM.docx]

**Supplementary data**

**Table S2. Clinical and biological characteristics of GBS neonatal infections**

| **Parameter** | **No. of cases** | |
| --- | --- | --- |
|  | **EOD** | **LOD** |
| **Male/female ratio** | **4/8** | **7/10** |
| **Age at time of onset (days), media (range)** | **0 (0-6)** | **19 (8-30)** |
| **Birth weight** |  |  |
| **>2500 g** | **12** | **15** |
| **Unknown** |  | **2** |
| **Gestational age** |  |  |
| **≥37 weeks** | **11** | **16** |
| **<37 weeks** | **1** | **1** |
| **Infection confirmed by culture** |  |  |
| **Sepsis** | **9*^c^*** | **15** |
| **Sepsis and meningitis** |  | **1** |
| **Bacteremia** | **1** |  |
| **Omphalitis** | **2** | **1*^d^*** |
| **Clinical signs of infection** |  |  |
| **Pneumonia** | **6** | **6** |
| **Meningitis** | **1** | **2** |
| **Pneumonia and meningitis** | **1** | **7** |
| **None** | **4** | **2** |
| **Targeted treatment*^a^*** |  |  |
| **PEN/MOX/VAN** | **0/1/0** | **2/0/1** |
| **PEN combining with other antibiotics** | **8** | **11** |
| **VAN combining with FEP** |  | **1** |
| **Empirical antibiotic treatment only*^b^*** | **3** | **2** |
| **Outcome** |  |  |
| **Cured** | **5** | **8** |
| **Improved** | **4** | **7** |
| **Dead** | **1*^e^*** | **1*^e^*** |
| **Unknown** | **2*^f^*** | **1*^f^*** |

EOD Early-onset disease LOD Late-onset disease PEN penicillin MOX moxalactam VAN vancomycin FEP cefepime

*a* Antibiotics against GBS were used after obtaining positive cultures.

*b* Patients died or were transferred to other hospitals for further therapy prior to obtaining the positive cultures.

*c* Including one case of sepsis caused by GBS and *Escherichia coli* infection simultaneously.

*d* The omphalitis was caused by GBS and *Klebsiella pneumonia* infection simultaneously.

*e* Patients died prior to obtaining the cultures.

*f* Unknown outcomes as patients were transferred to other hospitals for further therapy prior to obtaining the cultures.
